# Association of Medicaid Expansion With Postpartum Depression Treatment in Arkansas

**DOI:** 10.1001/jamahealthforum.2022.5603

**Published:** 2023-02-24

**Authors:** Maria W. Steenland, Amal N. Trivedi

**Affiliations:** 1 Population Studies and Training Center, Brown University, Providence, Rhode Island; 2 Department of Health Services, Policy, and Practice, Brown University School of Public Health, Providence, Rhode Island; 3 Providence VA Medical Center, Providence, Rhode Island

## Abstract

**Importance:**

Postpartum depression affects approximately 1 in every 8 postpartum individuals in the US. Antidepressant medication can effectively treat postpartum depression. However, gaps in postpartum insurance coverage after the end of Medicaid pregnancy coverage at 60 days postpartum may limit treatment uptake and decrease continuity of postpartum depression treatment.

**Objective:**

To examine the association of Medicaid expansion in Arkansas with postpartum antidepressant prescription fills and antidepressant continuation and supply during the first 6 months postpartum.

**Design, Setting, and Participants:**

Cohort study with a difference-in-differences analysis comparing persons with Medicaid and commercially financed childbirth using Arkansas’ All-Payer Claims Database (2013-2016). Analysis was completed between July 2021 and June 2022.

**Exposures:**

Medicaid-paid childbirth after January 1, 2014.

**Main Outcomes and Measures:**

Antidepressant medication prescription fills and the number of days of antidepressant supply in the early (first 60 days after childbirth) and the late (61 days to 6 months after childbirth) postpartum periods.

**Results:**

In this cohort study with a difference-in-differences analysis of 60 990 childbirths (mean [SD] birthing parent’s age, 27 [5.3] years; 22% Black, 7% Hispanic, 67% White individuals), 72% of births were paid for by Medicaid and 28% were paid for by a commercial payer. Before expansion, 4.2% of people with a Medicaid-paid birth filled an antidepressant prescription in the later postpartum period. Medicaid expansion was associated with a 4.6 percentage point (95% CI, 2.9-6.3) increase in the likelihood, or a relative change of 110%, in this outcome. Before expansion, among people with postpartum depression in the early postpartum period with a Medicaid-paid birth, 32.7% filled an antidepressant prescription in the later postpartum period, and had an average of 23 days of antidepressant prescription supply during the later postpartum period. Among people with early postpartum depression, Medicaid expansion increased the continuity of antidepressant treatment by 20.5 percentage points (95% CI, 14.1-26.9) and the number of days with antidepressant supply in the later postpartum period by 14.1 days (95% CI, 7.2-20.9).

**Conclusions and Relevance:**

Medicaid expansion in Arkansas was associated with an increase in postpartum antidepressant prescription fills, and an increase in antidepressant treatment continuity and medication supply in the period after Medicaid pregnancy-related eligibility ended.

## Key Points

**Question:**

Was Arkansas’ Medicaid expansion associated with an increase in postpartum antidepressant prescription fills in the first 6 months postpartum?

**Findings:**

In this cohort study using difference-in-differences analysis of 60 990 childbirths, Medicaid expansion was associated with increases in antidepressant prescription fills, psychotherapy visits, the continuity of antidepressant treatment, and the number of days with antidepressant supply in the later postpartum period (between 61 days and 6 months postpartum).

**Meaning:**

Postpartum insurance is an important driver of postpartum depression treatment; policy changes that extend pregnancy Medicaid, particularly in nonexpansion states, have the potential to increase treatment for postpartum depression.

## Teaser

This cohort study uses difference-in-differences analysis to examine the association of Medicaid expansion in Arkansas with postpartum antidepressant prescription fills and antidepressant continuation and supply during the first 6 months postpartum.

## Introduction

Postpartum depression affects approximately 1 in every 8 postpartum individuals in the US.^1^ The prevalence of postpartum depression is higher among people whose childbirth care is paid for by Medicaid (17%) than for people with a commercial payer during childbirth (10.1%). Postpartum depression is also more common among postpartum people who identify as American Indian/Alaska Native (22%), Asian/Pacific Islander (19.2%), and Black (18.2%) than among people identifying as White (11.4%).^1^

Postpartum depression is associated with reduced maternal quality of life, greater difficulty with social and partner relationships,^2^ increased likelihood of unemployment,^3^ and adverse child health outcomes such as behavioral problems.^4^ Postpartum depression and other mental health conditions can also result in adverse postpartum health outcomes. Psychiatric disease is the third most common reason for readmission within 6 weeks of childbirth^5^ and mental health conditions are the second leading cause of maternal mortality after 42 days postpartum.^6^ Notably, state maternal mortality review committees have estimated that 100% of maternal deaths due to mental health are preventable.^7^

Independently, studies have shown that antidepressant medication and psychotherapy are effective treatments for postpartum depression, resulting in statistically and clinically significant improvements in depression symptoms vs placebo and usual care groups, respectively.^8,9^ However, these therapies are often underused,^10^ particularly among Medicaid enrollees compared with people with private insurance, and Black compared with white individuals.^11,12,13^ Lack of access to health care, systemic racism, lack of access to child care, and transportation create barriers to postpartum depression treatment.^14^ Suggested policy solutions include expanding Medicaid to 1 year postpartum; however, little existing research has examined the role of postpartum insurance interruptions among Medicaid enrollees on treatment of postpartum depression.^15^

Despite the high prevalence of postpartum depression, prior to the Affordable Care Act, approximately 26% of new mothers with income under the poverty level were uninsured in the year after childbirth.^16^ People who lose health insurance postpartum are more likely to have difficulty obtaining care.^17^ By increasing income eligibility to 138% of the FPL for all adults, the ACA’s Medicaid expansion increased the continuity of insurance coverage after childbirth and the number of postpartum visits among low-income women.^16,18,19^ In the years after expansion (2015-2018), among people who had prenatal Medicaid coverage, 10% were uninsured postpartum in expansion states compared with 36% in nonexpansion states.^20^ Medicaid expansion decreased uninsurance among low-income adults with depression,^21^ and reduced psychological distress among low-income parents^22^ but was not associated with changes in postpartum depression symptoms or postpartum well-being.^23,24^ Research on the effects of expansion on treatment is limited, but 1 recent study^25^ found that Medicaid expansion in Oregon increased screening for and treatment of postpartum depression. In this article, we add to this literature by focusing on the effect of Arkansas’ Medicaid expansion to examine the association of Medicaid expansion with treatment for postpartum depression.

## Methods

### Arkansas’ Medicaid Expansion

Arkansas’ Medicaid expansion began on January 1, 2014. After expansion, Arkansas used a process referred to as the “private option,” to cover new Medicaid beneficiaries using Marketplace plans. After Medicaid expansion in Arkansas, income eligibility for parents increased from 16% of FPL in 2013 to 138% in 2014 and increased for nondisabled adults from 0% (2013) to 138% (2014). We selected Arkansas because of its very low-income eligibility limit for parental Medicaid before the ACA. Therefore, Arkansas’ postpartum population would be expected to benefit greatly from expansion.

### Study Design

This study used a difference-in-differences design to examine changes in receipt of treatment for postpartum depression among adults (aged ≥19 years) with Medicaid-financed births before (January-June 2013) and after expansion (January 2014-December 2015) relative to trends among postpartum adults with commercially financed births (including people with non-Medicaid marketplace plans). Before 2014, Medicaid postpartum coverage ended 60 days after childbirth at which point postpartum people had to meet more restrictive parental income eligibility limits (<17% of the FPL). After Medicaid expansion in Arkansas, postpartum people with Medicaid-financed births could qualify for extended Medicaid coverage beyond 60 days postpartum if their income was below 138% of the FPL. We assigned people with Medicaid-financed childbirth to the treatment group because people with Medicaid coverage during pregnancy must meet Medicaid’s pregnancy eligibility threshold of less than 214% FPL in Arkansas.^26^ People with Medicaid pregnancy coverage are therefore likely to meet income eligibility requirements for adult Medicaid coverage after expansion. The study analysis was completed between July 2021 and June 2022.

We followed the Strengthening the Reporting of Observational Studies in Epidemiology (STROBE) reporting guidelines.^27^ This study was considered not human participants research by Brown University’s Human Research Protection Program.

### Data and Study Population

The study population was composed of people who gave birth between 2013 and 2015 in Arkansas identified using Arkansas birth certificate records. We linked these birth certificate records to medical and pharmacy claims from Arkansas’ All Payers Claim Database (APCD) (2013-2016). The last name and date of birth of the birthing person was used to complete this linkage. Together, last name and date of birth uniquely identify approximately 96% of individuals in Arkansas giving birth in a given year.^28^ Because our linkage method did not rely on payer-specific IDs, the study data set included all claims during the 6 months after childbirth even if the patient switched from Medicaid to commercial coverage, or from 1 commercial payer or Marketplace plan to another. Individuals were assigned to the treatment group (Medicaid or commercially financed childbirth) based on the type of insurance they were enrolled in at the time of birth (Medicaid or a commercial payer).

The unit of analysis in this study was a childbirth (n = 60 990). The construction of the overall sample has been described in a previous manuscript.^19^ See eFigure 1 in Supplement 1 for a detailed description of the sample construction. We examined the study outcomes among the full population of childbirths in the sample (60 990) and among the subpopulation of people who gave birth and had an antidepressant prescription filled in the first 60 days postpartum (n = 4972). We focused on this subsample to isolate the effect of Medicaid expansion on the continuity of antidepressant medication use among people treated for postpartum depression.

Previous research has found that depression diagnoses are often not billed in claims data.^29,30^ Therefore, we chose to define the population of people with documented postpartum depression in the early postpartum period (ie, first 60 days postpartum) as anyone with an antidepressant prescription fill in the first 60 days postpartum. We tested this assumption by examining the share of people with an antidepressant prescription fill who had a depression diagnosis in their postpartum medical insurance claims.

### Outcomes

The primary outcomes were (1) at least 1 fill of an antidepressant medication; and (2) the number of days of antidepressant supply in the 6 months after childbirth. We used the dates of antidepressant prescription fills and the numbers of days supplied on each fill date to estimate the number of days between 61 days and 6 months postpartum during which the patient had antidepressant supply. The first outcome was examined during the first 60 days and separately between 61 days and 6 months postpartum. The second outcome was only examined between 61 days and 6 months postpartum. We refer to the first 60 days postpartum as the “early postpartum period,” and days 61 through 6 months postpartum as the “later postpartum period.”

The secondary outcomes were (1) psychotherapy examined separately during the early and late postpartum periods; and (2) diagnosis of depression during at least 1 outpatient visit in the 6 months after childbirth. Psychotherapy was included as a secondary outcome because it was used by less than 1% of the study population during the first 6 months postpartum. We used previously developed diagnosis and procedure code definitions to measure these outcomes.^11^

### Exposure Variables and Covariates

The primary exposure in the study analysis was an interaction term between Medicaid coverage at the time of childbirth and whether the delivery occurred after January 1, 2014. The study’s regression models all included controls for age, race and ethnicity, and education level as self-reported on the birth certificate. Self-reported race and ethnicity from birth certificate data were classified as Hispanic, non-Hispanic Black (hereafter Black), non-Hispanic White (hereafter White), and other or unknown race. Racial groups in the other category included Asian, Native American/Alaska Native, and Pacific Islander. Age was categorized into the following groups: aged 19 to 24, 25 to 30, 31 to 34, 35 to 50 years. Education was collapsed into a dichotomous indicator of completion of a college degree.

### Statistical Analysis

#### Regression Analysis

We used multivariable linear difference-in-differences regression models to examine the association between Medicaid expansion and the study outcomes. Each regression included an indicator variable for whether the birth was financed by Medicaid or a commercial payer, a postexpansion indicator for whether the birth took place after the start of Medicaid expansion, and an interaction term between Medicaid-financing and the postexpansion indicator. All births that took place between January 1, 2013, and June 30, 2013, were classified as having taken place in the preexpansion period. The 6-month postpartum period for people with births between July 1, 2013, and December 31, 2013, overlapped with the preexpansion and postexpansion periods. Therefore, this period was designated a transitional period, and was not included in the regression analyses. Births that took place in 2014 and 2015 were classified as the postexpansion period. As we had claims data through the end of 2016, we were able to follow the insurance and medical care of people with a childbirth during the full postperiod. Regression models adjusted for age, education, race and ethnicity, and county of residence with standard errors clustered at the individual level (9.2% of persons in our sample had more than 1 birth during the study period).

#### Tests for Parallel Pretrends

We examined whether the prepolicy trends in all of the study outcomes were parallel in the preexpansion period from January 1, 2013, to June 30, 2013. We also conducted difference-in-differences analyses where the outcome variables were sample characteristics (ie, total number of births, race and ethnicity, and mean age) to test for changes in the composition of persons with Medicaid or commercially financed births after Medicaid expansion.

## Results

The study sample included 60 990 births in Arkansas between 2013 and 2015. Medicaid was the payer for 71.7% (43 736) of births in the sample and 28.3% were paid for by a commercial payer (17 254) (Table 1). Persons with a Medicaid-paid birth were younger (mean age, 25.5 years) compared with people with a commercially paid birth (mean age, 29.4 years). Persons with a Medicaid birth were more likely to identify as Hispanic (4 734/43 736 [10.8%]) and Black (12 110/43 736 [27.6%]) than people with a commercially paid birth, among whom 3.0% (518/17 254) identified as Hispanic and 7.1% (1223/17 254) identified as Black (Table 1). A smaller percentage of people with a Medicaid-paid birth identified as White (58.0% or 25 495/43 736) compared with people with a commercially paid birth (86.2% or 14 832/17 254). Only 5.1% of people with a Medicaid paid birth had a college or higher level of education (2231/43 736) compared with 55.5% (9543/17 736) of people with a commercially paid birth (Table 1). Medicaid expansion was not associated with the total number of births paid for by each payer during the study period and did not result in large changes in the racial and ethnic composition of the study groups (eTable 1 in Supplement 1).

**Table 1.** Characteristics of Persons Giving Birth in Arkansas by the Source of Insurance Coverage at Delivery, 2013 to 2015^a^

Variable	No. (%)	
	Medicaid (n = 43 736)	Commercial (n = 17 254)
Age, mean (SD), y	25.5 (5.1)	29.4 (4.8)
19-24	22 057 (50.0)	2761 (16.0)
25-30	14 485 (32.8)	7682 (44.5)
31-35	4705 (10.7)	4272 (24.8)
36-50	2856 (6.5)	2539 (14.7)
Hispanic	4734 (10.8)	518 (3.0)
Non-Hispanic		
Black	12 110 (27.6)	1223 (7.1)
White	25 495 (58.0)	14 832 (86.2)
Other or unknown race^b^	1615 (3.7)	639 (3.7)
Less than college	41 397 (94.9)	7644 (44.5)
College or greater	2231 (5.1)	9543 (55.5)

^a^ Analysis of the Arkansas All Payer Claims Database.

^b^ Racial groups in the other category included Asian, Native American/Alaska Native, and Pacific Islander.

Among people with a Medicaid-paid birth who had an antidepressant prescription fill in the first 6 months postpartum, 34.8% (1872/5374) had a claim containing a diagnosis code for postpartum depression in the same time period. Among people with a commercially paid birth, this percentage was 27.0% (580/2174) (eFigure 2 in Supplement 1). Before Medicaid expansion, there was no difference in the trend in the study outcomes comparing between people with Medicaid and commercially paid childbirth (eTable 2 in Supplement 1).

**Figure 1.**
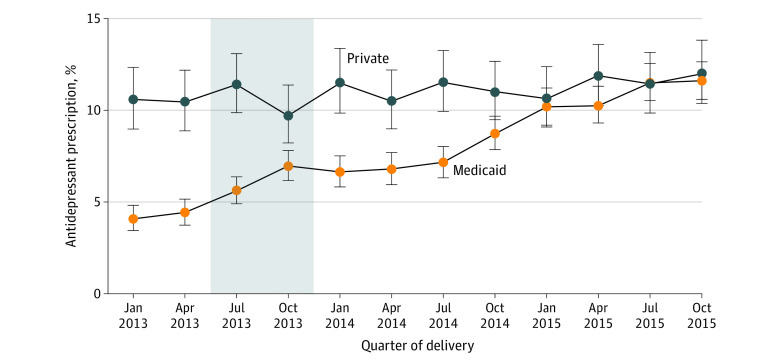
Percent of Postpartum People Who Filled a Prescription for an Antidepressant Between 61 Days and 6 Months Postpartum Among Persons With Medicaid and Commercially Financed Childbirth, 2013 to 2015 Analysis of the Arkansas All Payer Claims Database, 2013 to 2015. Each plotted point represents the percent of persons delivering in that quarter who filled an antidepressant prescription between 61 days and 6 months postpartum. Error bars represent the 95% CIs for each percent. The gray area represents the transition period, designated as such because the 6-month postpartum period for persons who gave birth between July and December 2013 overlapped only partially with the expansion period.

eFigure 3 in Supplement 1 plots the percent of persons who filled a prescription for an antidepressant before 60 days postpartum (the early postpartum period), and Figure 1 plots the percentage of persons who filled a prescription for an antidepressant between 61 days and 6 months postpartum (the later postpartum period). Before Medicaid expansion, between January and June 2013, 10.5% (95% CI, 9.4%-11.7%) of persons with commercial coverage at childbirth filled a prescription for an antidepressant in the later postpartum period and this percentage remained stable during 2014 and 2015 (Figure 1 and Table 2). Among persons covered by Medicaid at childbirth, before Medicaid expansion, 4.2% (95% CI, 3.8%-4.7%) filled an antidepressant prescription in the later postpartum period (Figure 1 and Table 2). The share of persons with Medicaid childbirth coverage who filled an antidepressant prescription in the later postpartum period increased to 7.3% in 2014 and further to 10.9% in 2015. Medicaid expansion was associated with a 4.6 percentage point (95% CI, 2.9-6.3) increase in the likelihood, or a relative change of 110%, of filling an antidepressant prescription in the later postpartum period (Table 2). Medicaid expansion was also associated with a smaller but statistically significant increase in antidepressant prescription fills in the first 60 days postpartum (difference-in-differences coefficient, 1.9; 95% CI, 0.2-3.7; or a 28% increase).

**Table 2.** Changes in Depression Treatment During the First 6 Months Postpartum Associated With Medicaid Expansion, 2013 to 2015^a^

Variable	Medicaid financed (n = 43 736), %			Commercially financed (n = 17 254), %			Adjusted difference-in-differences, % (95% CI)
	Preexpansion (January-June 2013)	Postexpansion year (2014-2015)	Unadjusted difference, preexpansion vs postexpansion	Preexpansion (January-June 2013)	Postexpansion year (2014-2015)	Unadjusted difference, preexpansion vs postexpansion, %	
Postpartum antidepressant prescription fills among all persons with a childbirth							
Antidepressant fill							
In the first 60 d postpartum	6.7	8.6	2.0	8.3	8.3	0	1.9 (0.2 to 3.7)
Between 61 d and 6 mos	4.2	9.3	5.1	10.5	11.0	0.5	4.6 (2.9 to 6.3)^b^
Postpartum psychotherapy visits among all persons with a childbirth							
Psychotherapy visit							
In the first 60 d postpartum	0.2	0.4	0.2	0.7	0.6	−0.1	0.2 (−0.1 to 0.5)
Between 61 d and 6 mo	0.2	1.0	0.7	1.3	1.2	−0.1	0.8 (0.5 to 1.2)^b^
Antidepressant prescription fills between 61 d and 6 mo postpartum among people with a fill in the first 60 d postpartum							
Antidepressant fill between 61 d and 6 mo	32.7	52.9	20.2	78.0	76.6	−1.3	20.5 (14.1 to 26.9)^b^
No. of days with supply between 61 d and 6 mo	23.0	41.0	17.9	71.6	72.5	0.8	14.1 (7.2 to 20.9)^b^

^a^ Source: Analysis of the Arkansas All Payer Claims Database, 2013 to 2015. Difference-in-differences regression models adjusted for age, education, race, and county of residence. Standard errors clustered at individual level.

^b^ *P* < .001.

**Figure 2.**
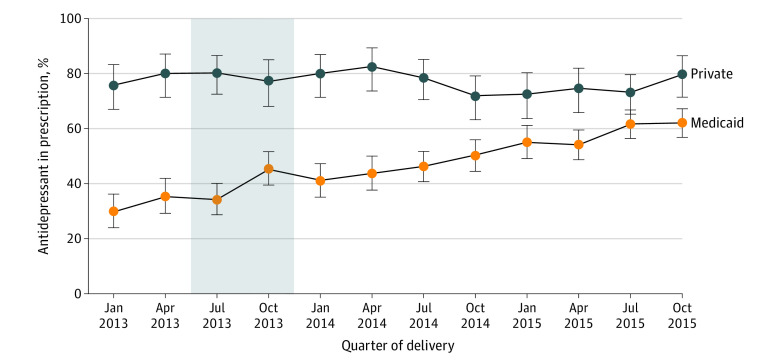
Percent of Postpartum People Who Filled an Antidepressant Prescription Between 61 Days and 6 Months Postpartum, Among Persons With Medicaid and Commercially Financed Childbirth Who Filled an Antidepressant Prescription in the First 60 Days Postpartum, 2013 to 2015 Analysis of the Arkansas All Payer Claims Database, 2013 to 2015. Each plotted point represents the percent of persons who delivered in that quarter and filled an antidepressant prescription in the first 60 days postpartum who went onto fill an antidepressant prescription between 61 days and 6 months postpartum. Error bars represent the 95% confidence interval for each percent. The gray area represents the transition period, designated as such because the 6-month postpartum period for persons who gave birth between July and December 2013 overlapped only partially with the expansion period.

Figure 2 plots the percent of persons who filled an antidepressant prescription between 61 days and 6 months postpartum in the subpopulation of people who filled a prescription for an antidepressant in the first 60 days postpartum (ie, people with depression in the early postpartum period). In this subpopulation, before Medicaid expansion, 78% (95% CI, 72.5-83.4) of people with a commercial payer filled an antidepressant prescription in the later postpartum period (Table 2). This percentage did not change after expansion (predifference postdifference in percent, −1.3; 95% CI, −7.5 to 4.8) (Table 2). Among Medicaid enrollees who received antidepressant therapy in the early postpartum period, 32.7% (95% CI, 28.4%-37.0%) filled an antidepressant prescription in the later postpartum period before Medicaid expansion. This percentage increased to 52.9% after Medicaid expansion. Medicaid expansion was associated with an increase in antidepressant prescription fills of 20.5 percentage points (95% CI, 14.1-26.9) among people with postpartum depression in the early postpartum period.

eFigures 4 and 5 in Supplement 1 plot the percentage of people with Medicaid and commercially paid births who had a psychotherapy visit in the early and late postpartum periods, respectively. Before expansion, 0.2% (15 of 6856) of persons with a Medicaid-paid birth and 1.3% (36 of 2723) of persons with a commercially paid birth had a psychotherapy visit in the later postpartum period. Medicaid expansion was associated with a 0.8 (95% CI, 0.5-1.2) percentage point increase in having at least 1 psychotherapy visit during the later postpartum period. Medicaid expansion was not associated with a change in receipt of psychotherapy in the early postpartum period (Table 2).

eFigure 6 in Supplement 1 plots the number of days of antidepressant supply between 61 days and 6 months postpartum among people with postpartum depression in the early postpartum period. In this subpopulation, before Medicaid expansion, on average people with a commercial payer had a 72-day antidepressant supply between 61 days and 6 months postpartum. Among people with Medicaid paid childbirth in the subpopulation with postpartum depression in the early postpartum period, antidepressant prescription supply in the later postpartum period was on average 23.0 days (95% CI, 20.7-25.4) before Medicaid expansion. Medicaid expansion was associated with an increase in antidepressant prescription supply in the later postpartum period of 14.1 days (95% CI, 7.2-20.9) among people with postpartum depression in the early postpartum period, a relative increase of 61%.

## Discussion

We investigated whether Medicaid expansion increased treatment for postpartum depression. We found that the percentage of people with Medicaid-financed childbirth who filled an antidepressant prescription in the later postpartum period increased after Medicaid expansion, substantially narrowing the gap in later postpartum depression treatment between people with commercial and Medicaid-financed childbirth. We also found that among people with postpartum depression in the early postpartum period, Medicaid expansion was associated with an increase in the continuity of treatment for postpartum depression and the number of days with antidepressant supply in the later postpartum period. Finally, we found that Medicaid expansion was associated with a small, but statistically significant, increase in receipt of psychotherapy in the later postpartum period.

A previous study conducted in Arkansas found that Medicaid expansion greatly increased the continuity of postpartum insurance coverage and the number of outpatient visits in the first six months postpartum.^19^ Our findings suggest that the large increases in postpartum insurance coverage available through parental Medicaid coverage after expansion allowed postpartum people with depression to continue using treatment initiated in the first 60 days postpartum. Continuity of treatment is important because antidepressants typically require up to 4 weeks of treatment before symptom improvement,^31^ and clinical guidelines recommend that patients should continue antidepressant treatment for 4 to 9 months after initial improvement.^32^

Previous studies^23,24^ using survey data from interviews conducted between 2 and 6 months postpartum have not found an association between Medicaid expansion nationally and symptoms of postpartum depression. Because treatment initiation is often delayed after a postpartum depression diagnosis, particularly for people with Medicaid coverage,^11^ it is possible surveys between 2 to 6 months postpartum may be too early to detect changes in depressive symptoms. However, evidence from the Oregon Health Insurance Experiment, a randomized study of Medicaid coverage in Oregon, found that Medicaid coverage in the general population resulted in large improvements in mental health outcomes including increased depression screening,^33^ increased depression treatment,^34^ and decreased depression,^35^ suggesting that mental health outcomes are very sensitive to expansions of insurance coverage.

These findings have important implications for health policy. First, Medicaid expansion likely improved postpartum depression treatment in expansion states. However, because postpartum uninsurance among people with a Medicaid-covered childbirth in Arkansas before expansion was higher than the average postpartum uninsurance rate among low-income women in expansion states before expansion,^18^ the average effect of Medicaid expansion on postpartum depression treatment may have been larger in Arkansas than in other expansion states.

Second, the American Rescue Plan Act of 2021 gives states an option to extend postpartum Medicaid coverage from 60 days to 12 months, beginning April 1, 2022.^36^ Medicaid postpartum extensions have already been implemented in 23 states and are pending implementation in an additional 11 states.^37^ The findings from this study suggest that 12-month extensions implemented in states that have not yet expanded Medicaid (eg, North Carolina, South Carolina, and Florida) may substantially increase the continuity of postpartum depression treatment for people with a Medicaid-paid childbirth. Medicaid extension policies in expansion states may have a more modest effect because they will increase eligibility for persons with higher income, some of whom would have already been insured postpartum.^20^ However, as postpartum depression affects 17% of people with pregnancy Medicaid coverage, Medicaid pregnancy extensions, even in expansion states, have the potential to have substantial benefits for population health, including improved quality of life, and reduced emergency department visits and hospital stays. Medicaid pregnancy coverage extensions may also reduce maternal mortality, particularly after 42 days postpartum, when maternal deaths due to mental health become a common cause of mortality.

### Limitations

This study has several limitations. First, Arkansas’ APCD began in 2013, just 1 year before the start of the state’s Medicaid expansion, which reduces the statistical power of our analysis comparing trends in the study outcomes before expansion. Second, we may have underestimated use of depression treatment among people without postpartum insurance because Arkansas’ APCD does not include self-pay outpatient care. Third, the Arkansas’ APCD does not include enrollees in self-insured plans. This omission could bias our estimates if the change in the trends in coverage and visits in this group differed from the trends in the employer-sponsored and commercially insured groups, or if Medicaid expansion substantially decreased churn between Medicaid pregnancy coverage and coverage through a self-paid plan in the postpartum period. Finally, we relied on receipt of an antidepressant fill within the first 60 days postpartum to identify people with postpartum depression since postpartum depression diagnosis is often not documented in claims data.^29,30^

## Conclusions

In this cohort study with a difference-in-differences analysis of 60 990 childbirths, we found that Medicaid expansion in Arkansas was associated with an increase in postpartum depression treatment. These results suggest that Medicaid expansion in states that have not yet implemented expansion, as well as 12-month Medicaid extensions under the American Rescue Plan Act, have the potential to increase treatment for postpartum depression and improve mental health among postpartum people.

## Notes

Supplement 1. eFigure 1. Study population eTable 1. Changes in the characteristics of persons with Medicaid and commercially paid childbirth associated with Medicaid expansion stratified by race, 2013-2015 eFigure 2: Percent with a depression diagnosis among people with and without an antidepressant prescription fill in the first 6-months postpartum by payer of delivery hospitalization, 2013-2015 eTable 2. Pre-Period Monthly trend Differences between persons with Medicaid and commercial coverage during childbirth, January-June 2013 eFigure 3. Percent of people who filled a prescription for an antidepressant in the first 60 days postpartum among persons with Medicaid and commercially financed childbirth, 2013-2015 eFigure 4. Percent of postpartum people with a psychotherapy visit in the first 60 days postpartum among persons with Medicaid and commercially financed childbirth, 2013-2015 eFigure 5. Percent of postpartum people with a psychotherapy visit between 61 days and six months postpartum among persons with Medicaid and commercially financed childbirth, 2013-2015 eFigure 6. Number of days with antidepressant supply between 61 days and 6 months postpartum, among persons with Medicaid and commercially financed childbirth who filled an antidepressant prescription in the first 60 days postpartum, 2013-2015

Supplement 2. Data Sharing Statement.
