# Supplementary material for: Association of Medicaid Expansion With Postpartum Depression Treatment in Arkansas
Source: JAMA Health Forum. 2023 Feb 24;4(2):e225603. doi: 10.1001/jamahealthforum.2022.5603 (PMC9958523; doi:10.1001/jamahealthforum.2022.5603)
Supplement: Supplement 1. — eFigure 1. Study population eTable 1. Changes in the characteristics of persons with Medicaid and commercially paid childbirth associated with Medicaid expansion stratified by race, 2013-2015 eFigure 2: Percent with a depression diagnosis among people with and without an antidepressant prescription fill in the first 6-months postpartum by payer of delivery hospitalization, 2013-2015 eTable 2. Pre-Period Monthly trend Differences between persons with Medicaid and commercial coverage during childbirth, January-June 2013 eFigure 3. Percent of people who filled a prescription for an antidepressant in the first 60 days postpartum among persons with Medicaid and commercially financed childbirth, 2013-2015 eFigure 4. Percent of postpartum people with a psychotherapy visit in the first 60 days postpartum among persons with Medicaid and commercially financed childbirth, 2013-2015 eFigure 5. Percent of postpartum people with a psychotherapy visit between 61 days and six months postpartum among persons with Medicaid and commercially financed childbirth, 2013-2015 eFigure 6. Number of days with antidepressant supply between 61 days and 6 months postpartum, among persons with Medicaid and commercially financed childbirth who filled an antidepressant prescription in the first 60 days postpartum, 2013-2015 [file jamahealthforum-e225603-s001.pdf]

Supplemental Online Content

Steenland MW, Trivedi AN. Association of Medicaid expansion with postpartum depression treatment in Arkansas. *JAMA Health Forum*. 2023;4(2):e225603. doi:10.1001/jamahealthforum.2022.5603

eFigure 1. Study population

eTable 1. Changes in the characteristics of persons with Medicaid and commercially paid childbirth associated with Medicaid expansion stratified by race, 2013-2015

eFigure 2: Percent with a depression diagnosis among people with and without an antidepressant prescription fill in the first 6-months postpartum by payer of delivery hospitalization, 2013-2015

eTable 2. Pre-Period Monthly trend Differences between persons with Medicaid and commercial coverage during childbirth, January-June 2013

eFigure 3. Percent of people who filled a prescription for an antidepressant in the first 60 days postpartum among persons with Medicaid and commercially financed childbirth, 2013-2015

eFigure 4. Percent of postpartum people with a psychotherapy visit in the first 60 days postpartum among persons with Medicaid and commercially financed childbirth, 2013-2015

eFigure 5. Percent of postpartum people with a psychotherapy visit between 61 days and six months postpartum among persons with Medicaid and commercially financed childbirth, 2013-2015

eFigure 6. Number of days with antidepressant supply between 61 days and 6 months postpartum, among persons with Medicaid and commercially financed childbirth who filled an antidepressant prescription in the first 60 days postpartum, 2013-2015

This supplemental material has been provided by the authors to give readers additional information about their work.

© 2023 Steenland et al. *JAMA Health Forum*.

eFigure 1: Study population:

Twenty six percent of births to insured adult Arkansas residents did not match to an enrollment record because they were covered in self-insured plans, which are not required to submit data to the APCD, or due to limitations of matching birth certificates to enrollment based on name and date of birth (i.e., name changes and typographical errors).

Sample inclusion flow-chart

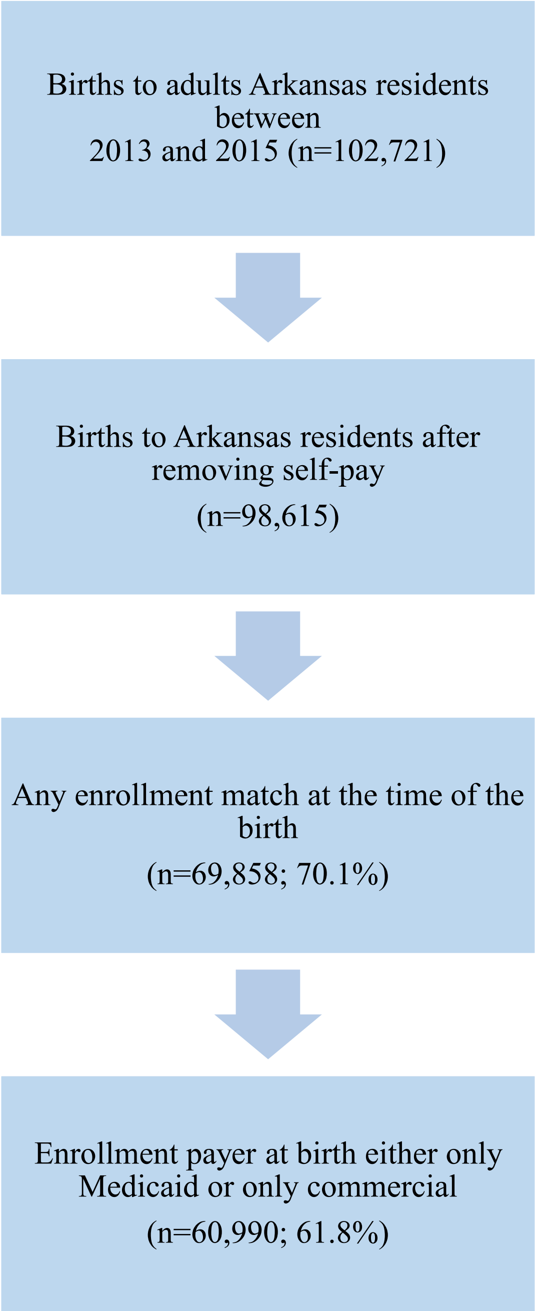

© 2023 Steenland et al. *JAMA Health Forum*.

eTable 1: Changes in the characteristics of persons with Medicaid and commercially paid childbirth associated with Medicaid expansion stratified by race, 2013—2015

	Medicaid January-June 2013	Medicaid 2014 and after	Medicaid Difference	Unadjusted difference between pre-and post-expansion	Commercial January-June 2013	Commercial 2014 and after	Commercial Difference	Unadjusted difference between pre-and post-expansion	Difference-in-Differences	
	Percent (95% CI)	Percent (95% CI)	Percentage Points (95% CI)	p-value	Percent (95% CI)	Percent (95% CI)	Percentage Points (95% CI)	p-value	Percentage Points (95% CI)	p-value
Age	25.3 (25.2, 25.4)	25.6 (25.6, 25.7)	0.3 (0.2, 0.5)	0.000	29.3 (29.2, 29.5)	29.6 (29.5, 29.6)	0.2 (0.0, 0.4)	0.034	0.1 (-0.1, 0.3)	0.373
Hispanic	12.2 (11.5, 13.0)	10.1 (9.8, 10.4)	-2.1 (-2.9, -1.3)	0.000	3.0 (2.3, 3.6)	3.1 (2.8, 3.4)	0.1 (-0.6, 0.8)	0.785	-2.0*** (-3.1, -1.0)	0.000
Black	27.6 (26.5, 28.6)	26.8 (26.3, 27.3)	-0.7 (-1.9, 0.4)	0.213	7.1 (6.1, 8.1)	6.8 (6.3, 7.3)	-0.3 (-1.4, 0.8)	0.574	-0.5 (-2.0, 0.9)	0.477
White	56.8 (55.6, 58.0)	59.2 (58.7, 59.8)	2.5 (1.2, 3.7)	0.000	86.0 (84.7, 87.3)	86.2 (85.5, 86.8)	0.2 (-1.2, 1.6)	0.787	2.1* (0.3, 3.9)	0.024
Total births	1,145 (1,144, 1,146)	1,214 (1,213, 1,215)	69 (66, 72)	0.000	455 (454, 456)	485 (484, 485)	29 (28, 31)	0.000	0.03 (-0.06, 0.12)	0.485

SOURCE Authors' analysis of the Arkansas All Payer Claims Database, 2013–15. NOTES: This analysis did not include education as an outcome because the coding of the education variable in Arkansas's birth certificate data changed between 2013 and 2014. Standard errors were clustered at the individual level. *p < 0.05 **p < 0.01 ***p < 0.001

© 2023 Steenland et al. *JAMA Health Forum*.

eFigure 2: Percent with a depression diagnosis among people with and without an antidepressant prescription fill in the first 6-months postpartum by payer of delivery hospitalization, 2013—2015

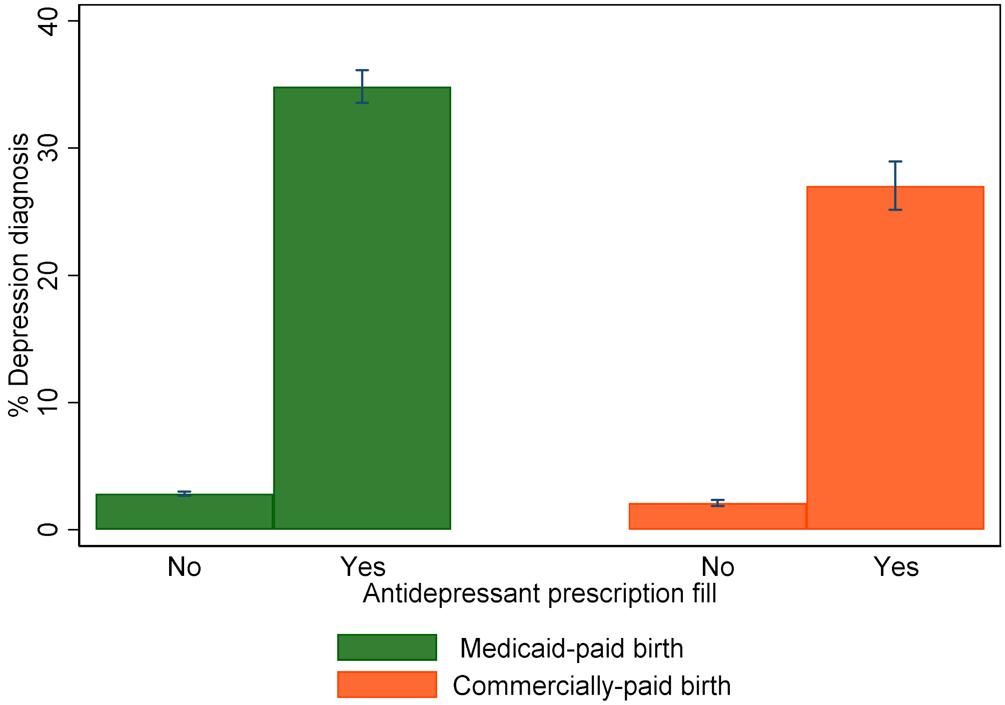

Note: Error bars represent the 95% confidence interval.

© 2023 Steenland et al. *JAMA Health Forum*.

eTable 2: Pre-Period Monthly trend Differences between persons with Medicaid and commercial coverage during childbirth, January – June 2013

	Full Sample				Persons who filled an antidepressant prescription in the first 60 days postpartum	
	Antidepressant fill in the first 60 days Postpartum	Antidepressant fill between 61 days and six months postpartum	Psychotherapy in the first 60 days Postpartum	Psychotherapy between 61 days and six months postpartum	Antidepressant fill between 61 days and six months postpartum	Number of days of prescription coverage between 61 days and six months postpartum
January – June 2013	Coefficient (95% CI)	Coefficient (95% CI)	Coefficient (95% CI)	Coefficient (95% CI)	Coefficient (95% CI)	Coefficient (95% CI)
Medicaid-paid birth	1.5 (-1.2, 4.2)	-3.6** (-6.1, -1.1)	-0.4 (-1.0, 0.3)	-1.5*** (-2.3, -0.7)	-35.0*** (-52.7, -17.2)	-34.5*** (-47.3, -21.6)
Trend	-0.2 (-0.8, 0.4)	0.3 (-0.3, 0.8)	-0.0 (-0.1, 0.1)	-0.1 (-0.2, 0.1)	2.5 (-1.4, 6.3)	2.7 (-0.1, 5.4)
Medicaid-paid birth*trend	-0.1 (-0.8, 0.6)	-0.1 (-0.8, 0.5)	-0.0 (-0.2, 0.1)	0.2 (-0.0, 0.3)	-0.3 (-4.7, 4.2)	-1.8 (-5.1, 1.4)
Constant	9.5** (3.3, 15.8)	11.9*** (6.1, 17.7)	0.3 (-1.1, 1.8)	1.0 (-0.8, 2.8)	61.1** (20.8, 101.5)	56.7*** (27.4, 85.9)
N	9,564	9,564	9,564	9,564	683	683

*p < 0.05 **p < 0.01 ***p < 0.001

Authors' analysis of the Arkansas All Payer Claims Database, January – June 2013

© 2023 Steenland et al. *JAMA Health Forum*.

eFigure 3: Percent of people who filled a prescription for an antidepressant in the first 60 days postpartum among persons with Medicaid and commercially financed childbirth, 2013—2015

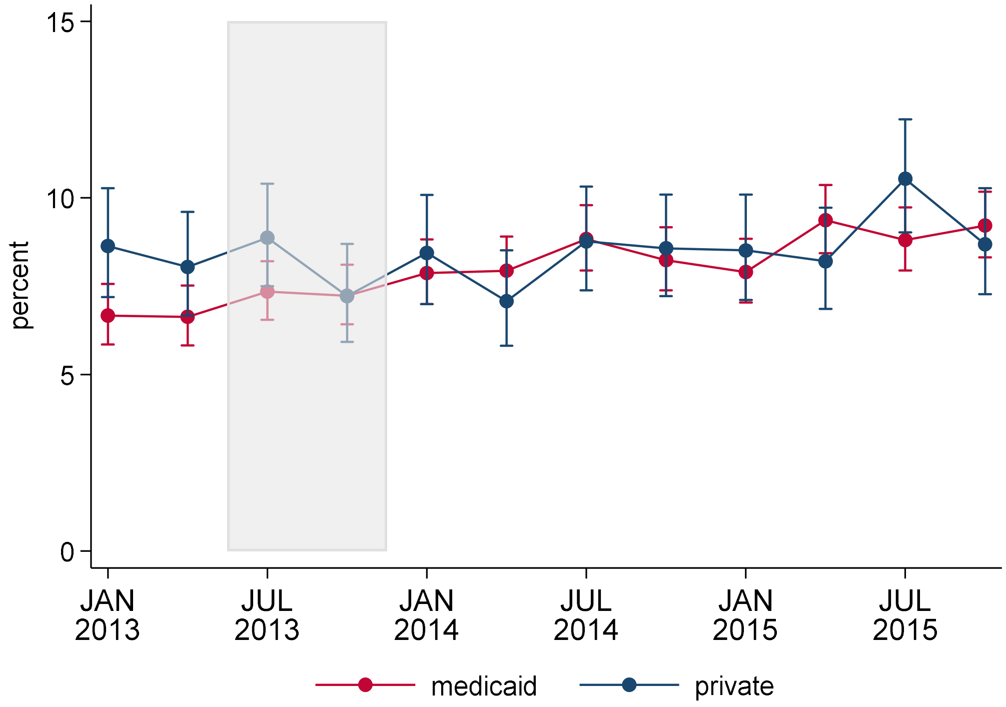

Authors' analysis of the Arkansas All Payer Claims Database, 2013–15. Each plotted point represents the percent of persons delivering in that quarter who filled an antidepressant prescription in the first 60 days postpartum. Error bars represent the 95% confidence interval for each percent. The grey area represents the transition period, designated as such because the six-month postpartum period for persons who gave birth between July and December 2013 overlapped only partially with the expansion period.

© 2023 Steenland et al. *JAMA Health Forum*.

eFigure 4: Percent of postpartum people with a psychotherapy visit in the first 60 days postpartum among persons with Medicaid and commercially financed childbirth, 2013—2015

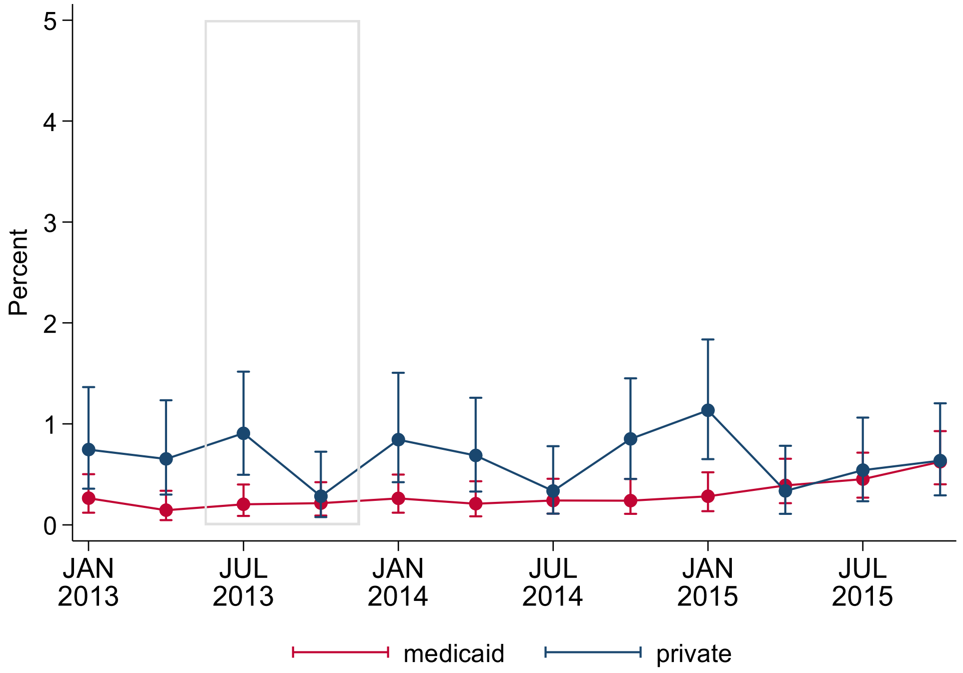

Authors' analysis of the Arkansas All Payer Claims Database, 2013–15. Each plotted point represents the percent of postpartum people delivering in that quarter with a psychotherapy visit in the first 60 days postpartum. Error bars represent the 95% confidence interval for each mean. The grey area represents the transition period, designated as such because the six-month postpartum period for persons who gave birth between July and December 2013 overlapped only partially with the expansion period.

© 2023 Steenland et al. *JAMA Health Forum*.

eFigure 5: Percent of postpartum people with a psychotherapy visit between 61 days and six months postpartum among persons with Medicaid and commercially financed childbirth, 2013—2015

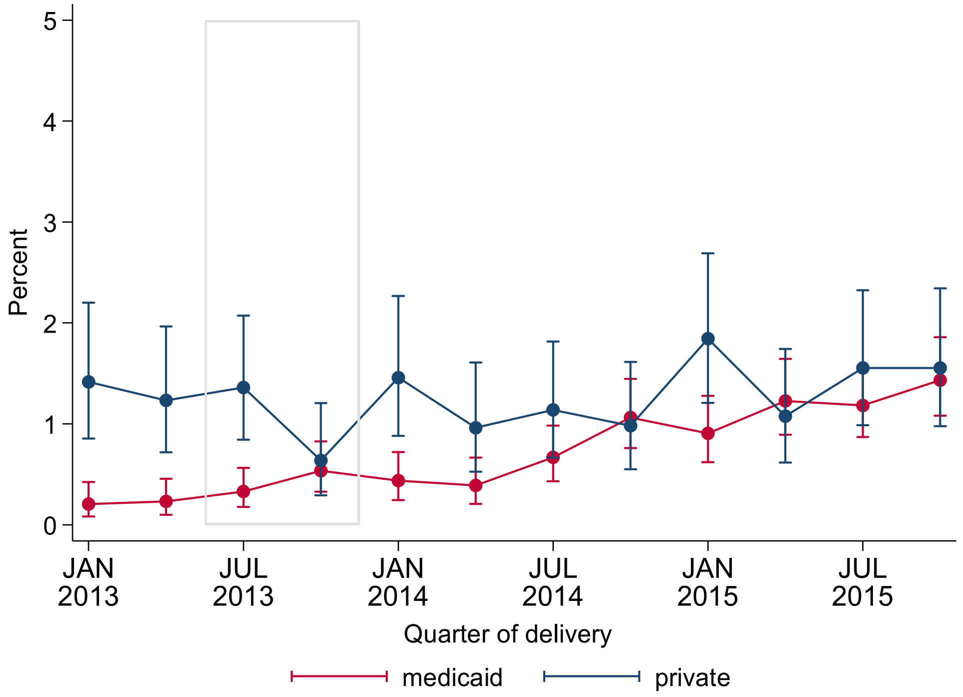

Authors' analysis of the Arkansas All Payer Claims Database, 2013–15. Each plotted point represents the percent of postpartum people delivering in that quarter with a psychotherapy visit between 61 days and six months postpartum among persons. Error bars represent the 95% confidence interval for each mean. The grey area represents the transition period, designated as such because the six-month postpartum period for persons who gave birth between July and December 2013 overlapped only partially with the expansion period.

© 2023 Steenland et al. *JAMA Health Forum*.

eFigure 6: Number of days with antidepressant supply between 61 days and 6 months postpartum, among persons with Medicaid and commercially financed childbirth who filled an antidepressant prescription in the first 60 days postpartum, 2013—2015

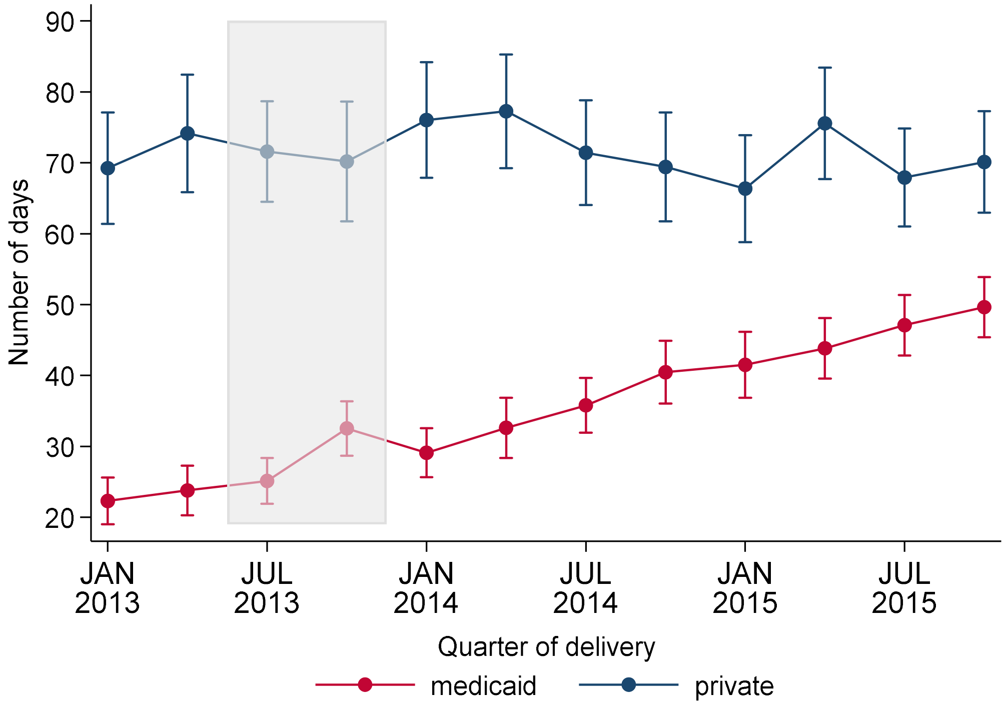

Authors' analysis of the Arkansas All Payer Claims Database, 2013–15. Each plotted point represents the mean number of days with antidepressant supply between 61 days and 6 months postpartum among persons who delivered in that quarter who filled an antidepressant prescription in the first 60 days postpartum. Error bars represent the 95% confidence interval for each mean. The grey area represents the transition period, designated as such because the six-month postpartum period for persons who gave birth between July and December 2013 overlapped only partially with the expansion period.

© 2023 Steenland et al. *JAMA Health Forum*.
