# Supplementary material for: Association of Medicaid Expansion With Postpartum Depression Treatment in Arkansas
Source: JAMA Health Forum. 2023 Feb 24;4(2):e225603. doi: 10.1001/jamahealthforum.2022.5603 (PMC9958523; doi:10.1001/jamahealthforum.2022.5603)
Supplement: Supplement 2. — Data Sharing Statement. [file jamahealthforum-e225603-s002.pdf]

Data Sharing Statement

Steenland. Association of Medicaid Expansion With Postpartum Depression Treatment in Arkansas. *JAMA Health Forum*. Published February 24, 2023.
doi:10.1001/jamahealthforum.2022.5603

Data
Data available: No

Additional Information
Explanation for why data not available: Arkansas' APCD data is available for purchase through the Arkansas Center for Health Improvement
